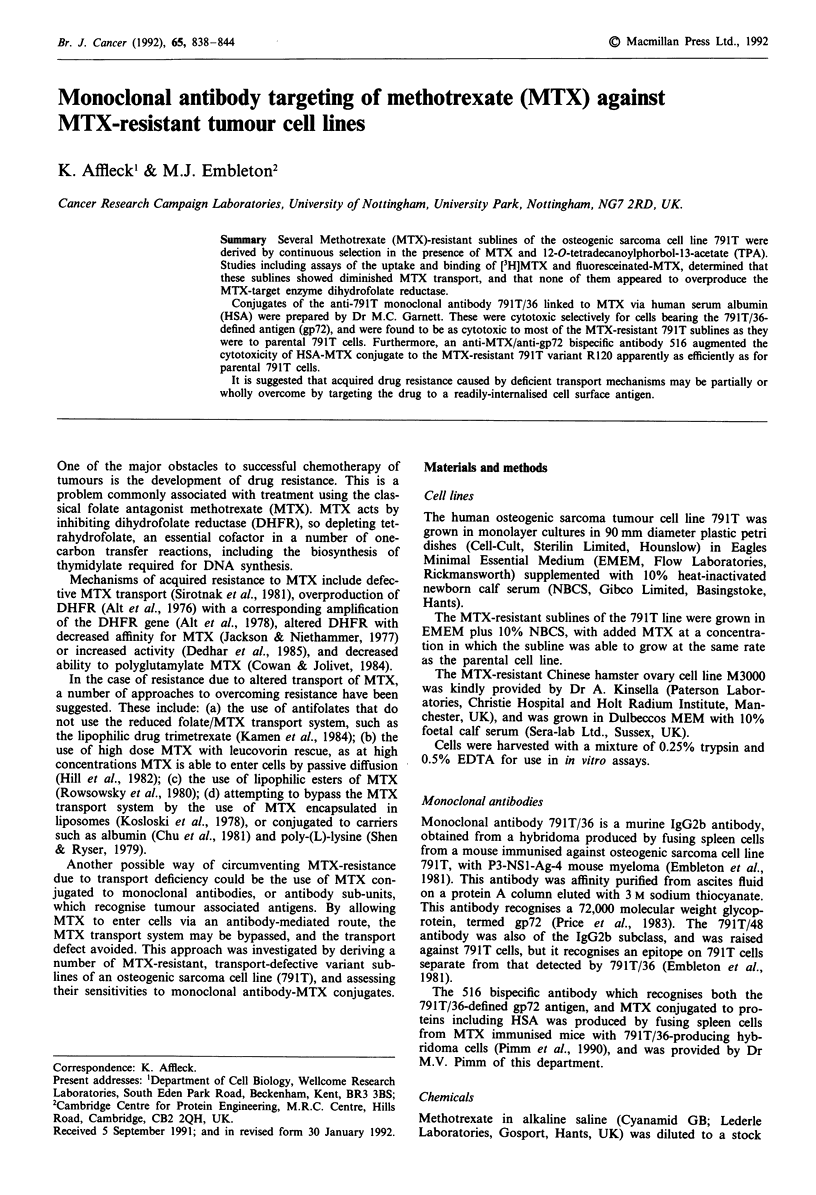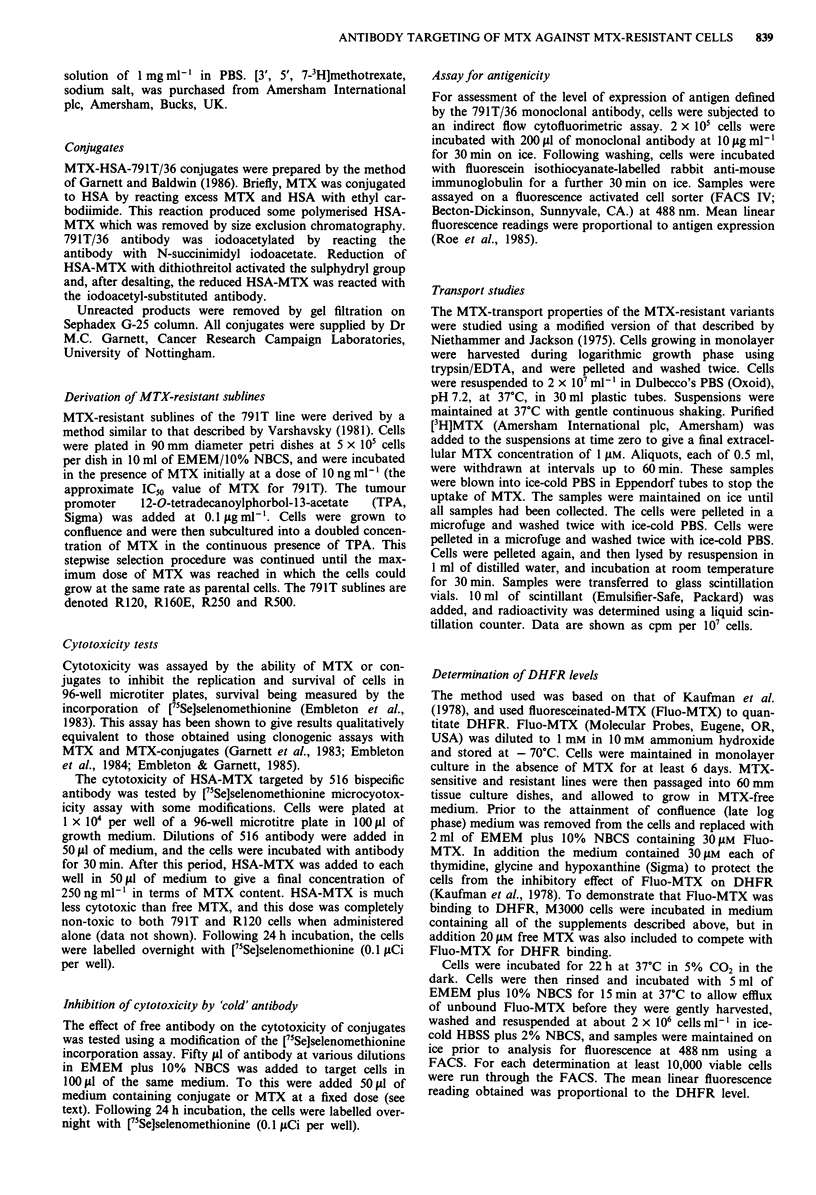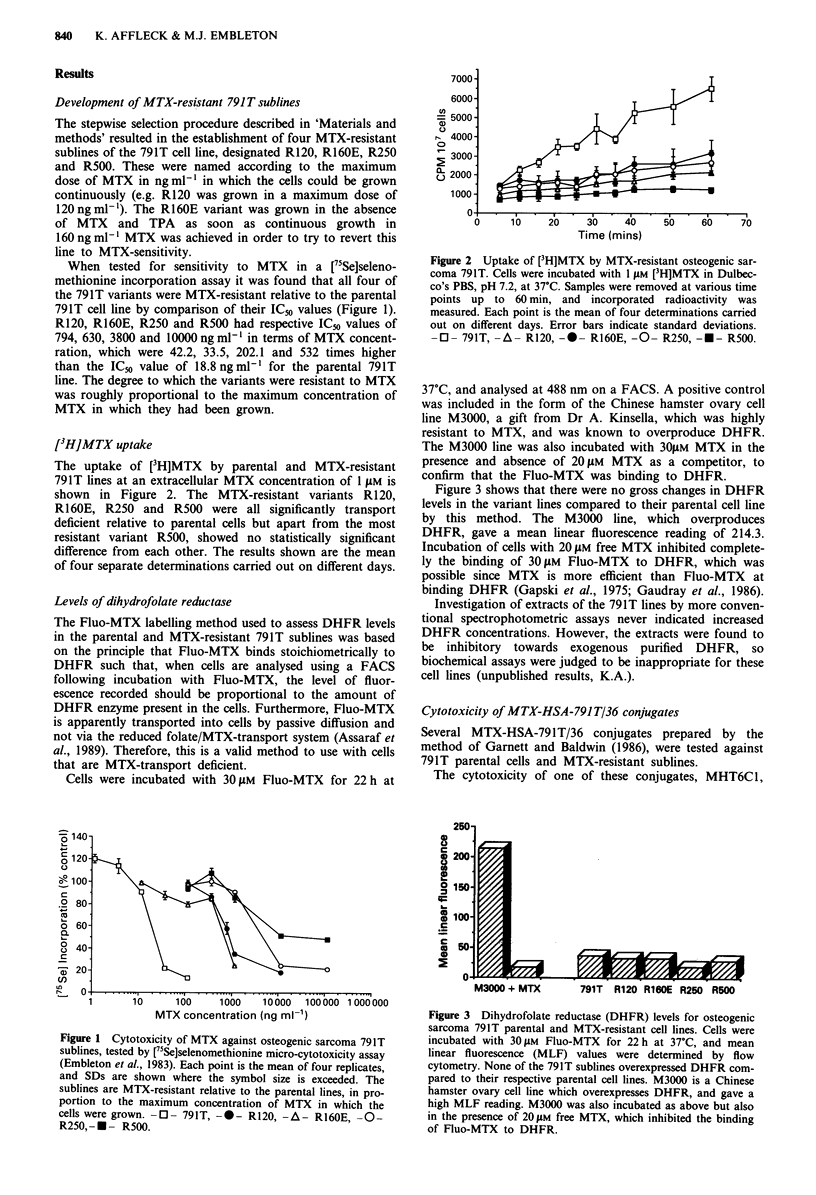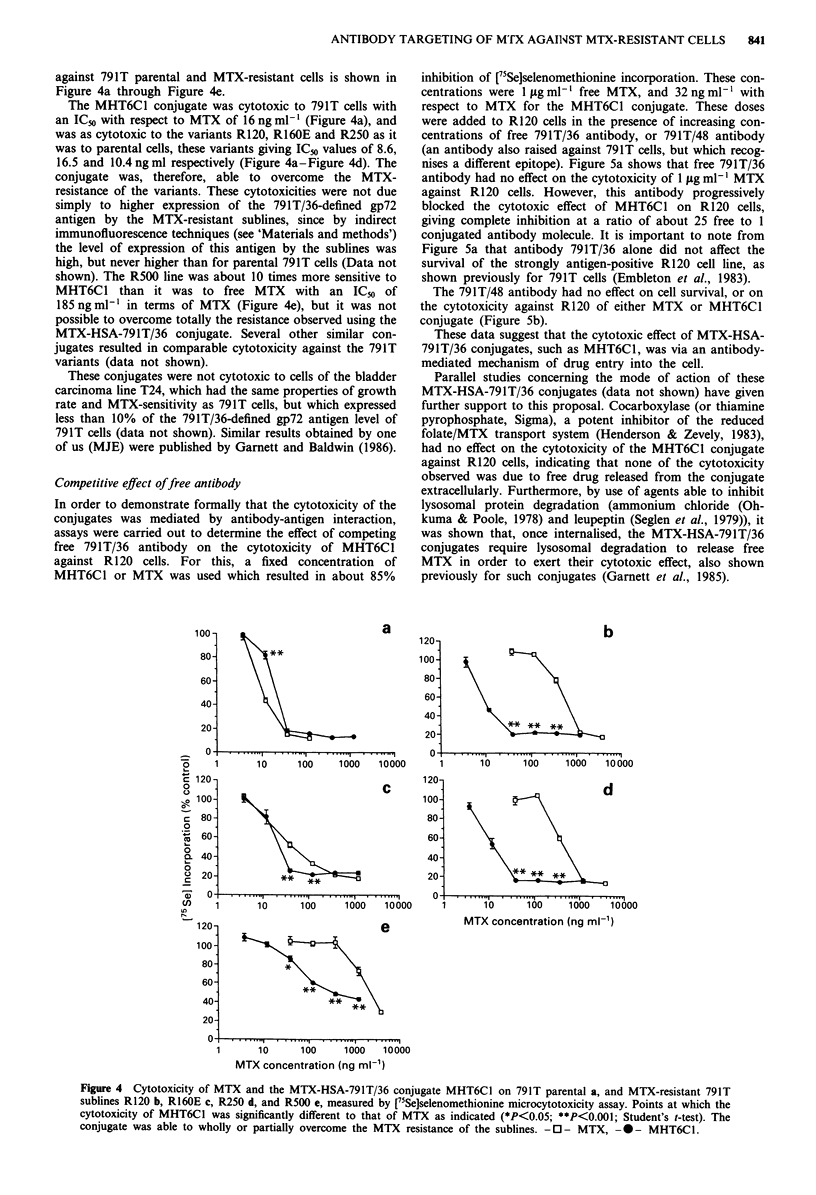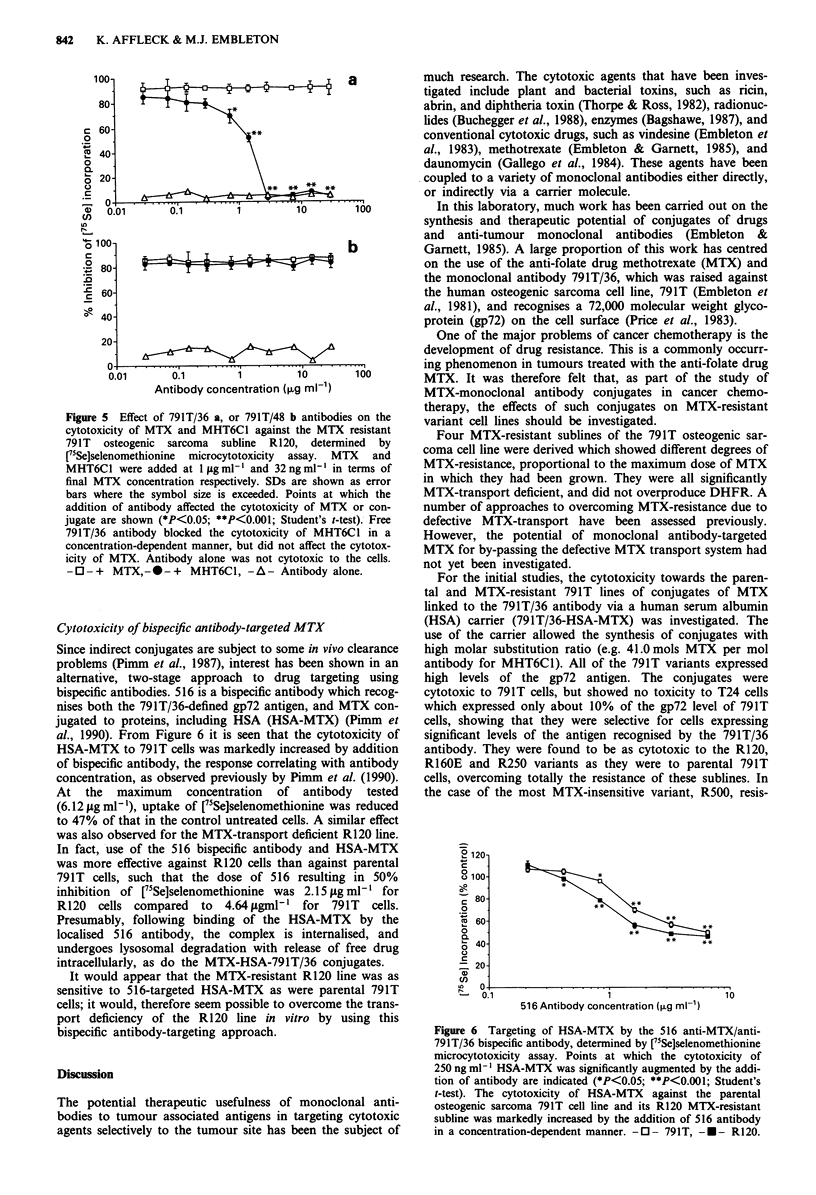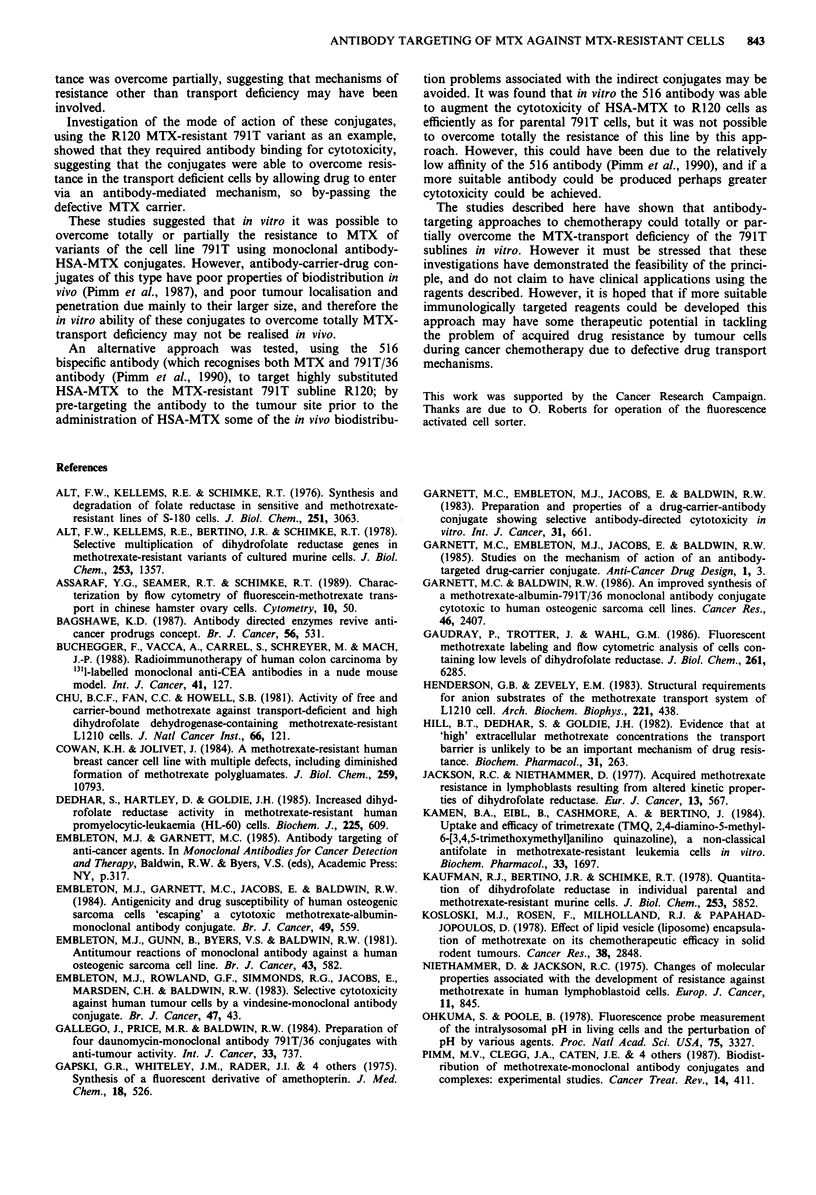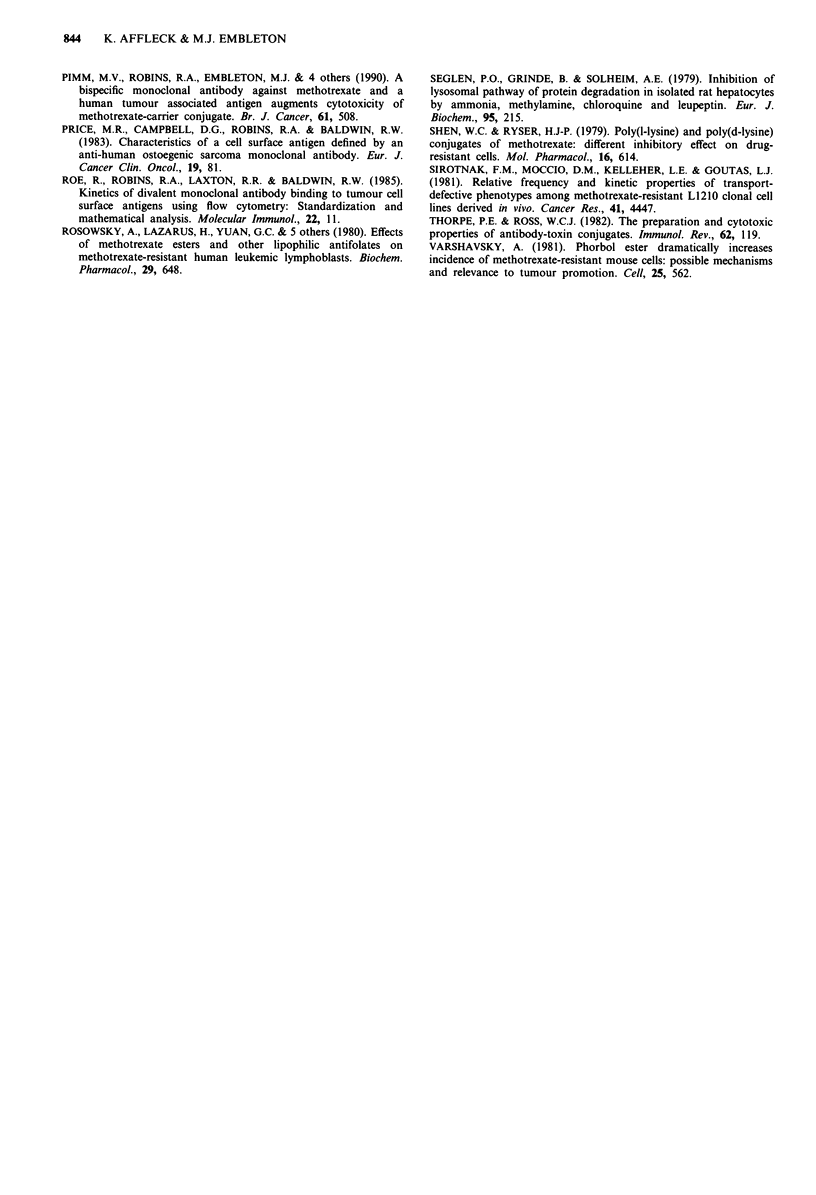# Monoclonal antibody targeting of methotrexate (MTX) against MTX-resistant tumour cell lines.

**DOI:** 10.1038/bjc.1992.178

**Published:** 1992-06

**Authors:** K. Affleck, M. J. Embleton

**Affiliations:** Cancer Research Campaign Laboratories, University of Nottingham, UK.

## Abstract

Several Methotrexate (MTX)-resistant sublines of the osteogenic sarcoma cell line 791T were derived by continuous selection in the presence of MTX and 12-O-tetradecanoylphorbol-13-acetate (TPA). Studies including assays of the uptake and binding of [3H]MTX and fluoresceinated-MTX, determined that these sublines showed diminished MTX transport, and that none of them appeared to overproduce the MTX-target enzyme dihydrofolate reductase. Conjugates of the anti-791T monoclonal antibody 791T/36 linked to MTX via human serum albumin (HSA) were prepared by Dr M.C. Garnett. These were cytotoxic selectively for cells bearing the 791T/36-defined antigen (gp72), and were found to be as cytotoxic to most of the MTX-resistant 791T sublines as they were to parental 791T cells. Furthermore, an anti-MTX/anti-gp72 bispecific antibody 516 augmented the cytotoxicity of HSA-MTX conjugate to the MTX-resistant 791T variant R120 apparently as efficiently as for parental 791T cells. It is suggested that acquired drug resistance caused by deficient transport mechanisms may be partially or wholly overcome by targeting the drug to a readily-internalised cell surface antigen.


Br. J. Cancer (1992), 65, 838–844 © Macmillan Press Ltd., 1992

### Monoclonal antibody targeting of methotrexate (MTX) against MTX-resistant tumour cell lines

K. Affleck[1] & M.J. Embleton[2]

*Cancer Research Campaign Laboratories, University of Nottingham, University Park, Nottingham, NG7 2RD, UK.*

**Summary** Several Methotrexate (MTX)-resistant sublines of the osteogenic sarcoma cell line 791T were derived by continuous selection in the presence of MTX and 12-*O*-tetradecanoylphorbol-13-acetate (TPA). Studies including assays of the uptake and binding of [$^3$H]MTX and fluoresceinated-MTX, determined that these sublines showed diminished MTX transport, and that none of them appeared to overproduce the MTX-target enzyme dihydrofolate reductase.

Conjugates of the anti-791T monoclonal antibody 791T/36 linked to MTX via human serum albumin (HSA) were prepared by Dr M.C. Garnett. These were cytotoxic selectively for cells bearing the 791T/36-defined antigen (gp72), and were found to be as cytotoxic to most of the MTX-resistant 791T sublines as they were to parental 791T cells. Furthermore, an anti-MTX/anti-gp72 bispecific antibody 516 augmented the cytotoxicity of HSA-MTX conjugate to the MTX-resistant 791T variant R120 apparently as efficiently as for parental 791T cells.

It is suggested that acquired drug resistance caused by deficient transport mechanisms may be partially or wholly overcome by targeting the drug to a readily-internalised cell surface antigen.

One of the major obstacles to successful chemotherapy of tumours is the development of drug resistance. This is a problem commonly associated with treatment using the classical folate antagonist methotrexate (MTX). MTX acts by inhibiting dihydrofolate reductase (DHFR), so depleting tetrahydrofolate, an essential cofactor in a number of one-carbon transfer reactions, including the biosynthesis of thymidylate required for DNA synthesis.

Mechanisms of acquired resistance to MTX include defective MTX transport (Sirotnak *et al.*, 1981), overproduction of DHFR (Alt *et al.*, 1976) with a corresponding amplification of the DHFR gene (Alt *et al.*, 1978), altered DHFR with decreased affinity for MTX (Jackson & Niethammer, 1977) or increased activity (Dedhar *et al.*, 1985), and decreased ability to polyglutamylate MTX (Cowan & Jolivet, 1984).

In the case of resistance due to altered transport of MTX, a number of approaches to overcoming resistance have been suggested. These include: (a) the use of antifolates that do not use the reduced folate/MTX transport system, such as the lipophilic drug trimetrexate (Kamen *et al.*, 1984); (b) the use of high dose MTX with leucovorin rescue, as at high concentrations MTX is able to enter cells by passive diffusion (Hill *et al.*, 1982); (c) the use of lipophilic esters of MTX (Rowsowsky *et al.*, 1980); (d) attempting to bypass the MTX transport system by the use of MTX encapsulated in liposomes (Kosloski *et al.*, 1978), or conjugated to carriers such as albumin (Chu *et al.*, 1981) and poly-(L)-lysine (Shen & Ryser, 1979).

Another possible way of circumventing MTX-resistance due to transport deficiency could be the use of MTX conjugated to monoclonal antibodies, or antibody sub-units, which recognise tumour associated antigens. By allowing MTX to enter cells via an antibody-mediated route, the MTX transport system may be bypassed, and the transport defect avoided. This approach was investigated by deriving a number of MTX-resistant, transport-defective variant sublines of an osteogenic sarcoma cell line (791T), and assessing their sensitivities to monoclonal antibody-MTX conjugates.

Correspondence: K. Affleck.

Present addresses: [1]Department of Cell Biology, Wellcome Research Laboratories, South Eden Park Road, Beckenham, Kent, BR3 3BS; [2]Cambridge Centre for Protein Engineering, M.R.C. Centre, Hills Road, Cambridge, CB2 2QH, UK.

Received 5 September 1991; and in revised form 30 January 1992.

### Materials and methods

### *Cell lines*

The human osteogenic sarcoma tumour cell line 791T was grown in monolayer cultures in 90 mm diameter plastic petri dishes (Cell-Cult, Sterilin Limited, Hounslow) in Eagles Minimal Essential Medium (EMEM, Flow Laboratories, Rickmansworth) supplemented with 10% heat-inactivated newborn calf serum (NBCS, Gibco Limited, Basingstoke, Hants).

The MTX-resistant sublines of the 791T line were grown in EMEM plus 10% NBCS, with added MTX at a concentration in which the subline was able to grow at the same rate as the parental cell line.

The MTX-resistant Chinese hamster ovary cell line M3000 was kindly provided by Dr A. Kinsella (Paterson Laboratories, Christie Hospital and Holt Radium Institute, Manchester, UK), and was grown in Dulbeccos MEM with 10% foetal calf serum (Sera-lab Ltd., Sussex, UK).

Cells were harvested with a mixture of 0.25% trypsin and 0.5% EDTA for use in *in vitro* assays.

### *Monoclonal antibodies*

Monoclonal antibody 791T/36 is a murine IgG2b antibody, obtained from a hybridoma produced by fusing spleen cells from a mouse immunised against osteogenic sarcoma cell line 791T, with P3-NS1-Ag-4 mouse myeloma (Embleton *et al.*, 1981). This antibody was affinity purified from ascites fluid on a protein A column eluted with 3 M sodium thiocyanate. This antibody recognises a 72,000 molecular weight glycoprotein, termed gp72 (Price *et al.*, 1983). The 791T/48 antibody was also of the IgG2b subclass, and was raised against 791T cells, but it recognises an epitope on 791T cells separate from that detected by 791T/36 (Embleton *et al.*, 1981).

The 516 bispecific antibody which recognises both the 791T/36-defined gp72 antigen, and MTX conjugated to proteins including HSA was produced by fusing spleen cells from MTX immunised mice with 791T/36-producing hybridoma cells (Pimm *et al.*, 1990), and was provided by Dr M.V. Pimm of this department.

### *Chemicals*

Methotrexate in alkaline saline (Cyanamid GB; Lederle Laboratories, Gosport, Hants, UK) was diluted to a stock solution of 1 mg $ml^{-1}$ in PBS. [3', 5', 7-$^3$H]methotrexate, sodium salt, was purchased from Amersham International plc, Amersham, Bucks, UK.

*Conjugates*

MTX-HSA-791T/36 conjugates were prepared by the method of Garnett and Baldwin (1986). Briefly, MTX was conjugated to HSA by reacting excess MTX and HSA with ethyl carbodiimide. This reaction produced some polymerised HSA-MTX which was removed by size exclusion chromatography. 791T/36 antibody was iodoacetylated by reacting the antibody with N-succinimidyl iodoacetate. Reduction of HSA-MTX with dithiothreitol activated the sulphydryl group and, after desalting, the reduced HSA-MTX was reacted with the iodoacetyl-substituted antibody.

Unreacted products were removed by gel filtration on Sephadex G-25 column. All conjugates were supplied by Dr M.C. Garnett, Cancer Research Campaign Laboratories, University of Nottingham.

*Derivation of MTX-resistant sublines*

MTX-resistant sublines of the 791T line were derived by a method similar to that described by Varshavsky (1981). Cells were plated in 90 mm diameter petri dishes at $5 \times 10^5$ cells per dish in 10 ml of EMEM/10% NBCS, and were incubated in the presence of MTX initially at a dose of 10 ng $ml^{-1}$ (the approximate $IC_{50}$ value of MTX for 791T). The tumour promoter 12-*O*-tetradecanoylphorbol-13-acetate (TPA, Sigma) was added at 0.1 μg $ml^{-1}$. Cells were grown to confluence and were then subcultured into a doubled concentration of MTX in the continuous presence of TPA. This stepwise selection procedure was continued until the maximum dose of MTX was reached in which the cells could grow at the same rate as parental cells. The 791T sublines are denoted R120, R160E, R250 and R500.

*Cytotoxicity tests*

Cytotoxicity was assayed by the ability of MTX or conjugates to inhibit the replication and survival of cells in 96-well microtiter plates, survival being measured by the incorporation of [$^{75}$Se]selenomethionine (Embleton *et al.*, 1983). This assay has been shown to give results qualitatively equivalent to those obtained using clonogenic assays with MTX and MTX-conjugates (Garnett *et al.*, 1983; Embleton *et al.*, 1984; Embleton & Garnett, 1985).

The cytotoxicity of HSA-MTX targeted by 516 bispecific antibody was tested by [$^{75}$Se]selenomethionine microcyotoxicity assay with some modifications. Cells were plated at $1 \times 10^4$ per well of a 96-well microtitre plate in 100 μl of growth medium. Dilutions of 516 antibody were added in 50 μl of medium, and the cells were incubated with antibody for 30 min. After this period, HSA-MTX was added to each well in 50 μl of medium to give a final concentration of 250 ng $ml^{-1}$ in terms of MTX content. HSA-MTX is much less cytotoxic than free MTX, and this dose was completely non-toxic to both 791T and R120 cells when administered alone (data not shown). Following 24 h incubation, the cells were labelled overnight with [$^{75}$Se]selenomethionine (0.1 μCi per well).

*Inhibition of cytotoxicity by 'cold' antibody*

The effect of free antibody on the cytotoxicity of conjugates was tested using a modification of the [$^{75}$Se]selenomethionine incorporation assay. Fifty μl of antibody at various dilutions in EMEM plus 10% NBCS was added to target cells in 100 μl of the same medium. To this were added 50 μl of medium containing conjugate or MTX at a fixed dose (see text). Following 24 h incubation, the cells were labelled overnight with [$^{75}$Se]selenomethionine (0.1 μCi per well).

*Assay for antigenicity*

For assessment of the level of expression of antigen defined by the 791T/36 monoclonal antibody, cells were subjected to an indirect flow cytofluorimetric assay. $2 \times 10^5$ cells were incubated with 200 μl of monoclonal antibody at 10 μg $ml^{-1}$ for 30 min on ice. Following washing, cells were incubated with fluorescein isothiocyanate-labelled rabbit anti-mouse immunoglobulin for a further 30 min on ice. Samples were assayed on a fluorescence activated cell sorter (FACS IV; Becton-Dickinson, Sunnyvale, CA.) at 488 nm. Mean linear fluorescence readings were proportional to antigen expression (Roe *et al.*, 1985).

*Transport studies*

The MTX-transport properties of the MTX-resistant variants were studied using a modified version of that described by Niethammer and Jackson (1975). Cells growing in monolayer were harvested during logarithmic growth phase using trypsin/EDTA, and were pelleted and washed twice. Cells were resuspended to $2 \times 10^7$ $ml^{-1}$ in Dulbecco's PBS (Oxoid), pH 7.2, at 37°C, in 30 ml plastic tubes. Suspensions were maintained at 37°C with gentle continuous shaking. Purified [$^3$H]MTX (Amersham International plc, Amersham) was added to the suspensions at time zero to give a final extracellular MTX concentration of 1 μM. Aliquots, each of 0.5 ml, were withdrawn at intervals up to 60 min. These samples were blown into ice-cold PBS in Eppendorf tubes to stop the uptake of MTX. The samples were maintained on ice until all samples had been collected. The cells were pelleted in a microfuge and washed twice with ice-cold PBS. Cells were pelleted in a microfuge and washed twice with ice-cold PBS. Cells were pelleted again, and then lysed by resuspension in 1 ml of distilled water, and incubation at room temperature for 30 min. Samples were transferred to glass scintillation vials. 10 ml of scintillant (Emulsifier-Safe, Packard) was added, and radioactivity was determined using a liquid scintillation counter. Data are shown as cpm per $10^7$ cells.

*Determination of DHFR levels*

The method used was based on that of Kaufman *et al.* (1978), and used fluoresceinated-MTX (Fluo-MTX) to quantitate DHFR. Fluo-MTX (Molecular Probes, Eugene, OR, USA) was diluted to 1 mM in 10 mM ammonium hydroxide and stored at − 70°C. Cells were maintained in monolayer culture in the absence of MTX for at least 6 days. MTX-sensitive and resistant lines were then passaged into 60 mm tissue culture dishes, and allowed to grow in MTX-free medium. Prior to the attainment of confluence (late log phase) medium was removed from the cells and replaced with 2 ml of EMEM plus 10% NBCS containing 30 μM Fluo-MTX. In addition the medium contained 30 μM each of thymidine, glycine and hypoxanthine (Sigma) to protect the cells from the inhibitory effect of Fluo-MTX on DHFR (Kaufman *et al.*, 1978). To demonstrate that Fluo-MTX was binding to DHFR, M3000 cells were incubated in medium containing all of the supplements described above, but in addition 20 μM free MTX was also included to compete with Fluo-MTX for DHFR binding.

Cells were incubated for 22 h at 37°C in 5% $CO_2$ in the dark. Cells were then rinsed and incubated with 5 ml of EMEM plus 10% NBCS for 15 min at 37°C to allow efflux of unbound Fluo-MTX before they were gently harvested, washed and resuspended at about $2 \times 10^6$ cells $ml^{-1}$ in ice-cold HBSS plus 2% NBCS, and samples were maintained on ice prior to analysis for fluorescence at 488 nm using a FACS. For each determination at least 10,000 viable cells were run through the FACS. The mean linear fluorescence reading obtained was proportional to the DHFR level.

### Results

### *Development of MTX-resistant 791T sublines*

The stepwise selection procedure described in 'Materials and methods' resulted in the establishment of four MTX-resistant sublines of the 791T cell line, designated R120, R160E, R250 and R500. These were named according to the maximum dose of MTX in ng $ml^{-1}$ in which the cells could be grown continuously (e.g. R120 was grown in a maximum dose of 120 ng $ml^{-1}$). The R160E variant was grown in the absence of MTX and TPA as soon as continuous growth in 160 ng $ml^{-1}$ MTX was achieved in order to try to revert this line to MTX-sensitivity.

When tested for sensitivity to MTX in a [$^{75}$Se]selenomethionine incorporation assay it was found that all four of the 791T variants were MTX-resistant relative to the parental 791T cell line by comparison of their $IC_{50}$ values (Figure 1). R120, R160E, R250 and R500 had respective $IC_{50}$ values of 794, 630, 3800 and 10000 ng $ml^{-1}$ in terms of MTX concentration, which were 42.2, 33.5, 202.1 and 532 times higher than the $IC_{50}$ value of 18.8 ng $ml^{-1}$ for the parental 791T line. The degree to which the variants were resistant to MTX was roughly proportional to the maximum concentration of MTX in which they had been grown.

### *[$^{3}$H]MTX uptake*

The uptake of [$^{3}$H]MTX by parental and MTX-resistant 791T lines at an extracellular MTX concentration of 1 $\mu$M is shown in Figure 2. The MTX-resistant variants R120, R160E, R250 and R500 were all significantly transport deficient relative to parental cells but apart from the most resistant variant R500, showed no statistically significant difference from each other. The results shown are the mean of four separate determinations carried out on different days.

### *Levels of dihydrofolate reductase*

The Fluo-MTX labelling method used to assess DHFR levels in the parental and MTX-resistant 791T sublines was based on the principle that Fluo-MTX binds stoichiometrically to DHFR such that, when cells are analysed using a FACS following incubation with Fluo-MTX, the level of fluorescence recorded should be proportional to the amount of DHFR enzyme present in the cells. Furthermore, Fluo-MTX is apparently transported into cells by passive diffusion and not via the reduced folate/MTX-transport system (Assaraf *et al.*, 1989). Therefore, this is a valid method to use with cells that are MTX-transport deficient.

Cells were incubated with 30 $\mu$M Fluo-MTX for 22 h at

**Figure 2** Uptake of [$^{3}$H]MTX by MTX-resistant osteogenic sarcoma 791T. Cells were incubated with 1 $\mu$M [$^{3}$H]MTX in Dulbecco's PBS, pH 7.2, at 37°C. Samples were removed at various time points up to 60 min, and incorporated radioactivity was measured. Each point is the mean of four determinations carried out on different days. Error bars indicate standard deviations. -□- 791T, -△- R120, -●- R160E, -○- R250, -■- R500.

37°C, and analysed at 488 nm on a FACS. A positive control was included in the form of the Chinese hamster ovary cell line M3000, a gift from Dr A. Kinsella, which was highly resistant to MTX, and was known to overproduce DHFR. The M3000 line was also incubated with 30$\mu$M MTX in the presence and absence of 20 $\mu$M MTX as a competitor, to confirm that the Fluo-MTX was binding to DHFR.

Figure 3 shows that there were no gross changes in DHFR levels in the variant lines compared to their parental cell line by this method. The M3000 line, which overproduces DHFR, gave a mean linear fluorescence reading of 214.3. Incubation of cells with 20 $\mu$M free MTX inhibited completely the binding of 30 $\mu$M Fluo-MTX to DHFR, which was possible since MTX is more efficient than Fluo-MTX at binding DHFR (Gapski *et al.*, 1975; Gaudray *et al.*, 1986).

Investigation of extracts of the 791T lines by more conventional spectrophotometric assays never indicated increased DHFR concentrations. However, the extracts were found to be inhibitory towards exogenous purified DHFR, so biochemical assays were judged to be inappropriate for these cell lines (unpublished results, K.A.).

### *Cytotoxicity of MTX-HSA-791T/36 conjugates*

Several MTX-HSA-791T/36 conjugates prepared by the method of Garnett and Baldwin (1986), were tested against 791T parental cells and MTX-resistant sublines.

The cytotoxicity of one of these conjugates, MHT6C1,

**Figure 1** Cytotoxicity of MTX against osteogenic sarcoma 791T sublines, tested by [$^{75}$Se]selenomethionine micro-cytotoxicity assay (Embleton *et al.*, 1983). Each point is the mean of four replicates, and SDs are shown where the symbol size is exceeded. The sublines are MTX-resistant relative to the parental lines, in proportion to the maximum concentration of MTX in which the cells were grown. -□- 791T, -●- R120, -△- R160E, -○- R250, -■- R500.

**Figure 3** Dihydrofolate reductase (DHFR) levels for osteogenic sarcoma 791T parental and MTX-resistant cell lines. Cells were incubated with 30 $\mu$M Fluo-MTX for 22 h at 37°C, and mean linear fluorescence (MLF) values were determined by flow cytometry. None of the 791T sublines overexpressed DHFR compared to their respective parental cell lines. M3000 is a Chinese hamster ovary cell line which overexpresses DHFR, and gave a high MLF reading. M3000 was also incubated as above but also in the presence of 20 $\mu$M free MTX, which inhibited the binding of Fluo-MTX to DHFR.

against 791T parental and MTX-resistant cells is shown in Figure 4a through Figure 4e.

The MHT6C1 conjugate was cytotoxic to 791T cells with an $IC_{50}$ with respect to MTX of 16 ng ml$^{-1}$ (Figure 4a), and was as cytotoxic to the variants R120, R160E and R250 as it was to parental cells, these variants giving $IC_{50}$ values of 8.6, 16.5 and 10.4 ng ml respectively (Figure 4a–Figure 4d). The conjugate was, therefore, able to overcome the MTX-resistance of the variants. These cytotoxicities were not due simply to higher expression of the 791T/36-defined gp72 antigen by the MTX-resistant sublines, since by indirect immunofluorescence techniques (see 'Materials and methods') the level of expression of this antigen by the sublines was high, but never higher than for parental 791T cells (Data not shown). The R500 line was about 10 times more sensitive to MHT6C1 than it was to free MTX with an $IC_{50}$ of 185 ng ml$^{-1}$ in terms of MTX (Figure 4e), but it was not possible to overcome totally the resistance observed using the MTX-HSA-791T/36 conjugate. Several other similar conjugates resulted in comparable cytotoxicity against the 791T variants (data not shown).

These conjugates were not cytotoxic to cells of the bladder carcinoma line T24, which had the same properties of growth rate and MTX-sensitivity as 791T cells, but which expressed less than 10% of the 791T/36-defined gp72 antigen level of 791T cells (data not shown). Similar results obtained by one of us (MJE) were published by Garnett and Baldwin (1986).

*Competitive effect of free antibody*

In order to demonstrate formally that the cytotoxicity of the conjugates was mediated by antibody-antigen interaction, assays were carried out to determine the effect of competing free 791T/36 antibody on the cytotoxicity of MHT6C1 against R120 cells. For this, a fixed concentration of MHT6C1 or MTX was used which resulted in about 85% inhibition of [$^{75}$Se]selenomethionine incorporation. These concentrations were 1 μg ml$^{-1}$ free MTX, and 32 ng ml$^{-1}$ with respect to MTX for the MHT6C1 conjugate. These doses were added to R120 cells in the presence of increasing concentrations of free 791T/36 antibody, or 791T/48 antibody (an antibody also raised against 791T cells, but which recognises a different epitope). Figure 5a shows that free 791T/36 antibody had no effect on the cytotoxicity of 1 μg ml$^{-1}$ MTX against R120 cells. However, this antibody progressively blocked the cytotoxic effect of MHT6C1 on R120 cells, giving complete inhibition at a ratio of about 25 free to 1 conjugated antibody molecule. It is important to note from Figure 5a that antibody 791T/36 alone did not affect the survival of the strongly antigen-positive R120 cell line, as shown previously for 791T cells (Embleton *et al.*, 1983).

The 791T/48 antibody had no effect on cell survival, or on the cytotoxicity against R120 of either MTX or MHT6C1 conjugate (Figure 5b).

These data suggest that the cytotoxic effect of MTX-HSA-791T/36 conjugates, such as MHT6C1, was via an antibody-mediated mechanism of drug entry into the cell.

Parallel studies concerning the mode of action of these MTX-HSA-791T/36 conjugates (data not shown) have given further support to this proposal. Cocarboxylase (or thiamine pyrophosphate, Sigma), a potent inhibitor of the reduced folate/MTX transport system (Henderson & Zevely, 1983), had no effect on the cytotoxicity of the MHT6C1 conjugate against R120 cells, indicating that none of the cytotoxicity observed was due to free drug released from the conjugate extracellularly. Furthermore, by use of agents able to inhibit lysosomal protein degradation (ammonium chloride (Ohkuma & Poole, 1978) and leupeptin (Seglen *et al.*, 1979)), it was shown that, once internalised, the MTX-HSA-791T/36 conjugates require lysosomal degradation to release free MTX in order to exert their cytotoxic effect, also shown previously for such conjugates (Garnett *et al.*, 1985).

**Figure 4** Cytotoxicity of MTX and the MTX-HSA-791T/36 conjugate MHT6C1 on 791T parental **a**, and MTX-resistant 791T sublines R120 **b**, R160E **c**, R250 **d**, and R500 **e**, measured by [$^{75}$Se]selenomethionine microcytotoxicity assay. Points at which the cytotoxicity of MHT6C1 was significantly different to that of MTX as indicated (*$P<0.05$; **$P<0.001$; Student's *t*-test). The conjugate was able to wholly or partially overcome the MTX resistance of the sublines. –□– MTX, –●– MHT6C1.

**Figure 5** Effect of 791T/36 **a**, or 791T/48 **b** antibodies on the cytotoxicity of MTX and MHT6C1 against the MTX resistant 791T osteogenic sarcoma subline R120, determined by [$^{75}$Se]selenomethionine microcytotoxicity assay. MTX and MHT6C1 were added at 1 μg ml$^{-1}$ and 32 ng ml$^{-1}$ in terms of final MTX concentration respectively. SDs are shown as error bars where the symbol size is exceeded. Points at which the addition of antibody affected the cytotoxicity of MTX or conjugate are shown (*$P<0.05$; **$P<0.001$; Student's *t*-test). Free 791T/36 antibody blocked the cytotoxicity of MHT6C1 in a concentration-dependent manner, but did not affect the cytotoxicity of MTX. Antibody alone was not cytotoxic to the cells. -□-+ MTX,-●-+ MHT6C1, -△- Antibody alone.

### *Cytotoxicity of bispecific antibody-targeted MTX*

Since indirect conjugates are subject to some *in vivo* clearance problems (Pimm *et al.*, 1987), interest has been shown in an alternative, two-stage approach to drug targeting using bispecific antibodies. 516 is a bispecific antibody which recognises both the 791T/36-defined gp72 antigen, and MTX conjugated to proteins, including HSA (HSA-MTX) (Pimm *et al.*, 1990). From Figure 6 it is seen that the cytotoxicity of HSA-MTX to 791T cells was markedly increased by addition of bispecific antibody, the response correlating with antibody concentration, as observed previously by Pimm *et al.* (1990). At the maximum concentration of antibody tested (6.12 μg ml$^{-1}$), uptake of [$^{75}$Se]selenomethionine was reduced to 47% of that in the control untreated cells. A similar effect was also observed for the MTX-transport deficient R120 line. In fact, use of the 516 bispecific antibody and HSA-MTX was more effective against R120 cells than against parental 791T cells, such that the dose of 516 resulting in 50% inhibition of [$^{75}$Se]selenomethionine was 2.15 μg ml$^{-1}$ for R120 cells compared to 4.64 μgml$^{-1}$ for 791T cells. Presumably, following binding of the HSA-MTX by the localised 516 antibody, the complex is internalised, and undergoes lysosomal degradation with release of free drug intracellularly, as do the MTX-HSA-791T/36 conjugates.

It would appear that the MTX-resistant R120 line was as sensitive to 516-targeted HSA-MTX as were parental 791T cells; it would, therefore seem possible to overcome the transport deficiency of the R120 line *in vitro* by using this bispecific antibody-targeting approach.

### Discussion

The potential therapeutic usefulness of monoclonal antibodies to tumour associated antigens in targeting cytotoxic agents selectively to the tumour site has been the subject of much research. The cytotoxic agents that have been investigated include plant and bacterial toxins, such as ricin, abrin, and diphtheria toxin (Thorpe & Ross, 1982), radionuclides (Buchegger *et al.*, 1988), enzymes (Bagshawe, 1987), and conventional cytotoxic drugs, such as vindesine (Embleton *et al.*, 1983), methotrexate (Embleton & Garnett, 1985), and daunomycin (Gallego *et al.*, 1984). These agents have been coupled to a variety of monoclonal antibodies either directly, or indirectly via a carrier molecule.

In this laboratory, much work has been carried out on the synthesis and therapeutic potential of conjugates of drugs and anti-tumour monoclonal antibodies (Embleton & Garnett, 1985). A large proportion of this work has centred on the use of the anti-folate drug methotrexate (MTX) and the monoclonal antibody 791T/36, which was raised against the human osteogenic sarcoma cell line, 791T (Embleton *et al.*, 1981), and recognises a 72,000 molecular weight glycoprotein (gp72) on the cell surface (Price *et al.*, 1983).

One of the major problems of cancer chemotherapy is the development of drug resistance. This is a commonly occurring phenomenon in tumours treated with the anti-folate drug MTX. It was therefore felt that, as part of the study of MTX-monoclonal antibody conjugates in cancer chemotherapy, the effects of such conjugates on MTX-resistant variant cell lines should be investigated.

Four MTX-resistant sublines of the 791T osteogenic sarcoma cell line were derived which showed different degrees of MTX-resistance, proportional to the maximum dose of MTX in which they had been grown. They were all significantly MTX-transport deficient, and did not overproduce DHFR. A number of approaches to overcoming MTX-resistance due to defective MTX-transport have been assessed previously. However, the potential of monoclonal antibody-targeted MTX for by-passing the defective MTX transport system had not yet been investigated.

For the initial studies, the cytotoxicity towards the parental and MTX-resistant 791T lines of conjugates of MTX linked to the 791T/36 antibody via a human serum albumin (HSA) carrier (791T/36-HSA-MTX) was investigated. The use of the carrier allowed the synthesis of conjugates with high molar substitution ratio (e.g. 41.0 mols MTX per mol antibody for MHT6C1). All of the 791T variants expressed high levels of the gp72 antigen. The conjugates were cytotoxic to 791T cells, but showed no toxicity to T24 cells which expressed only about 10% of the gp72 level of 791T cells, showing that they were selective for cells expressing significant levels of the antigen recognised by the 791T/36 antibody. They were found to be as cytotoxic to the R120, R160E and R250 variants as they were to parental 791T cells, overcoming totally the resistance of these sublines. In the case of the most MTX-insensitive variant, R500, resis-

**Figure 6** Targeting of HSA-MTX by the 516 anti-MTX/anti-791T/36 bispecific antibody, determined by [$^{75}$Se]selenomethionine microcytotoxicity assay. Points at which the cytotoxicity of 250 ng ml$^{-1}$ HSA-MTX was significantly augmented by the addition of antibody are indicated (*$P<0.05$; **$P<0.001$; Student's *t*-test). The cytotoxicity of HSA-MTX against the parental osteogenic sarcoma 791T cell line and its R120 MTX-resistant subline was markedly increased by the addition of 516 antibody in a concentration-dependent manner. -□- 791T, -■- R120.

tance was overcome partially, suggesting that mechanisms of resistance other than transport deficiency may have been involved.

Investigation of the mode of action of these conjugates, using the R120 MTX-resistant 791T variant as an example, showed that they required antibody binding for cytotoxicity, suggesting that the conjugates were able to overcome resistance in the transport deficient cells by allowing drug to enter via an antibody-mediated mechanism, so by-passing the defective MTX carrier.

These studies suggested that *in vitro* it was possible to overcome totally or partially the resistance to MTX of variants of the cell line 791T using monoclonal antibody-HSA-MTX conjugates. However, antibody-carrier-drug conjugates of this type have poor properties of biodistribution *in vivo* (Pimm *et al.*, 1987), and poor tumour localisation and penetration due mainly to their larger size, and therefore the *in vitro* ability of these conjugates to overcome totally MTX-transport deficiency may not be realised *in vivo*.

An alternative approach was tested, using the 516 bispecific antibody (which recognises both MTX and 791T/36 antibody (Pimm *et al.*, 1990), to target highly substituted HSA-MTX to the MTX-resistant 791T subline R120; by pre-targeting the antibody to the tumour site prior to the administration of HSA-MTX some of the *in vivo* biodistribution problems associated with the indirect conjugates may be avoided. It was found that *in vitro* the 516 antibody was able to augment the cytotoxicity of HSA-MTX to R120 cells as efficiently as for parental 791T cells, but it was not possible to overcome totally the resistance of this line by this approach. However, this could have been due to the relatively low affinity of the 516 antibody (Pimm *et al.*, 1990), and if a more suitable antibody could be produced perhaps greater cytotoxicity could be achieved.

The studies described here have shown that antibody-targeting approaches to chemotherapy could totally or partially overcome the MTX-transport deficiency of the 791T sublines *in vitro*. However it must be stressed that these investigations have demonstrated the feasibility of the principle, and do not claim to have clinical applications using the ragents described. However, it is hoped that if more suitable immunologically targeted reagents could be developed this approach may have some therapeutic potential in tackling the problem of acquired drug resistance by tumour cells during cancer chemotherapy due to defective drug transport mechanisms.

This work was supported by the Cancer Research Campaign. Thanks are due to O. Roberts for operation of the fluorescence activated cell sorter.

### References

ALT, F.W., KELLEMS, R.E. & SCHIMKE, R.T. (1976). Synthesis and degradation of folate reductase in sensitive and methotrexate-resistant lines of S-180 cells. *J. Biol. Chem.*, **251**, 3063.

ALT, F.W., KELLEMS, R.E., BERTINO, J.R. & SCHIMKE, R.T. (1978). Selective multiplication of dihydrofolate reductase genes in methotrexate-resistant variants of cultured murine cells. *J. Biol. Chem.*, **253**, 1357.

ASSARAF, Y.G., SEAMER, R.T. & SCHIMKE, R.T. (1989). Characterization by flow cytometry of fluorescein-methotrexate transport in chinese hamster ovary cells. *Cytometry*, **10**, 50.

BAGSHAWE, K.D. (1987). Antibody directed enzymes revive anti-cancer prodrugs concept. *Br. J. Cancer*, **56**, 531.

BUCHEGGER, F., VACCA, A., CARREL, S., SCHREYER, M. & MACH, J.-P. (1988). Radioimmunotherapy of human colon carcinoma by $^{131}$I-labelled monoclonal anti-CEA antibodies in a nude mouse model. *Int. J. Cancer*, **41**, 127.

CHU, B.C.F., FAN, C.C. & HOWELL, S.B. (1981). Activity of free and carrier-bound methotrexate against transport-deficient and high dihydrofolate dehydrogenase-containing methotrexate-resistant L1210 cells. *J. Natl Cancer Inst.*, **66**, 121.

COWAN, K.H. & JOLIVET, J. (1984). A methotrexate-resistant human breast cancer cell line with multiple defects, including diminished formation of methotrexate polygluamates. *J. Biol. Chem.*, **259**, 10793.

DEDHAR, S., HARTLEY, D. & GOLDIE, J.H. (1985). Increased dihydrofolate reductase activity in methotrexate-resistant human promyelocytic-leukaemia (HL-60) cells. *Biochem. J.*, **225**, 609.

EMBLETON, M.J. & GARNETT, M.C. (1985). Antibody targeting of anti-cancer agents. In *Monoclonal Antibodies for Cancer Detection and Therapy*, Baldwin, R.W. & Byers, V.S. (eds), Academic Press: NY, p.317.

EMBLETON, M.J., GARNETT, M.C., JACOBS, E. & BALDWIN, R.W. (1984). Antigenicity and drug susceptibility of human osteogenic sarcoma cells 'escaping' a cytotoxic methotrexate-albumin-monoclonal antibody conjugate. *Br. J. Cancer*, **49**, 559.

EMBLETON, M.J., GUNN, B., BYERS, V.S. & BALDWIN, R.W. (1981). Antitumour reactions of monoclonal antibody against a human osteogenic sarcoma cell line. *Br. J. Cancer*, **43**, 582.

EMBLETON, M.J., ROWLAND, G.F., SIMMONDS, R.G., JACOBS, E., MARSDEN, C.H. & BALDWIN, R.W. (1983). Selective cytotoxicity against human tumour cells by a vindesine-monoclonal antibody conjugate. *Br. J. Cancer*, **47**, 43.

GALLEGO, J., PRICE, M.R. & BALDWIN, R.W. (1984). Preparation of four daunomycin-monoclonal antibody 791T/36 conjugates with anti-tumour activity. *Int. J. Cancer*, **33**, 737.

GAPSKI, G.R., WHITELEY, J.M., RADER, J.I. & 4 others (1975). Synthesis of a fluorescent derivative of amethopterin. *J. Med. Chem.*, **18**, 526.

GARNETT, M.C., EMBLETON, M.J., JACOBS, E. & BALDWIN, R.W. (1983). Preparation and properties of a drug-carrier-antibody conjugate showing selective antibody-directed cytotoxicity *in vitro*. *Int. J. Cancer*, **31**, 661.

GARNETT, M.C., EMBLETON, M.J., JACOBS, E. & BALDWIN, R.W. (1985). Studies on the mechanism of action of an antibody-targeted drug-carrier conjugate. *Anti-Cancer Drug Design*, **1**, 3.

GARNETT, M.C. & BALDWIN, R.W. (1986). An improved synthesis of a methotrexate-albumin-791T/36 monoclonal antibody conjugate cytotoxic to human osteogenic sarcoma cell lines. *Cancer Res.*, **46**, 2407.

GAUDRAY, P., TROTTER, J. & WAHL, G.M. (1986). Fluorescent methotrexate labeling and flow cytometric analysis of cells containing low levels of dihydrofolate reductase. *J. Biol. Chem.*, **261**, 6285.

HENDERSON, G.B. & ZEVELY, E.M. (1983). Structural requirements for anion substrates of the methotrexate transport system of L1210 cell. *Arch. Biochem. Biophys.*, **221**, 438.

HILL, B.T., DEDHAR, S. & GOLDIE, J.H. (1982). Evidence that at 'high' extracellular methotrexate concentrations the transport barrier is unlikely to be an important mechanism of drug resistance. *Biochem. Pharmacol.*, **31**, 263.

JACKSON, R.C. & NIETHAMMER, D. (1977). Acquired methotrexate resistance in lymphoblasts resulting from altered kinetic properties of dihydrofolate reductase. *Eur. J. Cancer*, **13**, 567.

KAMEN, B.A., EIBL, B., CASHMORE, A. & BERTINO, J. (1984). Uptake and efficacy of trimetrexate (TMQ, 2,4-diamino-5-methyl-6-[3,4,5-trimethoxymethyl]anilino quinazoline), a non-classical antifolate in methotrexate-resistant leukemia cells *in vitro*. *Biochem. Pharmacol.*, **33**, 1697.

KAUFMAN, R.J., BERTINO, J.R. & SCHIMKE, R.T. (1978). Quantitation of dihydrofolate reductase in individual parental and methotrexate-resistant murine cells. *J. Biol. Chem.*, **253**, 5852.

KOSLOSKI, M.J., ROSEN, F., MILHOLLAND, R.J. & PAPAHADJOPOULOS, D. (1978). Effect of lipid vesicle (liposome) encapsulation of methotrexate on its chemotherapeutic efficacy in solid rodent tumours. *Cancer Res.*, **38**, 2848.

NIETHAMMER, D. & JACKSON, R.C. (1975). Changes of molecular properties associated with the development of resistance against methotrexate in human lymphoblastoid cells. *Europ. J. Cancer*, **11**, 845.

OHKUMA, S. & POOLE, B. (1978). Fluorescence probe measurement of the intralysosomal pH in living cells and the perturbation of pH by various agents. *Proc. Natl Acad. Sci. USA*, **75**, 3327.

PIMM, M.V., CLEGG, J.A., CATEN, J.E. & 4 others (1987). Biodistribution of methotrexate-monoclonal antibody conjugates and complexes: experimental studies. *Cancer Treat. Rev.*, **14**, 411.

PIMM, M.V., ROBINS, R.A., EMBLETON, M.J. & 4 others (1990). A bispecific monoclonal antibody against methotrexate and a human tumour associated antigen augments cytotoxicity of methotrexate-carrier conjugate. *Br. J. Cancer*, **61**, 508.

PRICE, M.R., CAMPBELL, D.G., ROBINS, R.A. & BALDWIN, R.W. (1983). Characteristics of a cell surface antigen defined by an anti-human ostoegenic sarcoma monoclonal antibody. *Eur. J. Cancer Clin. Oncol.*, **19**, 81.

ROE, R., ROBINS, R.A., LAXTON, R.R. & BALDWIN, R.W. (1985). Kinetics of divalent monoclonal antibody binding to tumour cell surface antigens using flow cytometry: Standardization and mathematical analysis. *Molecular Immunol.*, **22**, 11.

ROSOWSKY, A., LAZARUS, H., YUAN, G.C. & 5 others (1980). Effects of methotrexate esters and other lipophilic antifolates on methotrexate-resistant human leukemic lymphoblasts. *Biochem. Pharmacol.*, **29**, 648.

SEGLEN, P.O., GRINDE, B. & SOLHEIM, A.E. (1979). Inhibition of lysosomal pathway of protein degradation in isolated rat hepatocytes by ammonia, methylamine, chloroquine and leupeptin. *Eur. J. Biochem.*, **95**, 215.

SHEN, W.C. & RYSER, H.J-P. (1979). Poly(l-lysine) and poly(d-lysine) conjugates of methotrexate: different inhibitory effect on drug-resistant cells. *Mol. Pharmacol.*, **16**, 614.

SIROTNAK, F.M., MOCCIO, D.M., KELLEHER, L.E. & GOUTAS, L.J. (1981). Relative frequency and kinetic properties of transport-defective phenotypes among methotrexate-resistant L1210 clonal cell lines derived *in vivo*. *Cancer Res.*, **41**, 4447.

THORPE, P.E. & ROSS, W.C.J. (1982). The preparation and cytotoxic properties of antibody-toxin conjugates. *Immunol. Rev.*, **62**, 119.

VARSHAVSKY, A. (1981). Phorbol ester dramatically increases incidence of methotrexate-resistant mouse cells: possible mechanisms and relevance to tumour promotion. *Cell*, **25**, 562.